# Association of leuko-glycemic index with mortality in ICU patients with Acute kidney injury: A retrospective multicenter cohort study

**DOI:** 10.1371/journal.pone.0350811

**Published:** 2026-06-04

**Authors:** Xuejin Ye, Sheng Chen, Lin Guo, Xiaohan Ma, Lingling Wu, Yiwen Li, Ting Zhang, Peng Jiang, Hongjun Gao

**Affiliations:** 1 Graduate School, Guangxi University of Chinese Medicine, Nanning, Guangxi, China; 2 The Second Affiliated Hospital, Guangxi University of Chinese Medicine, Nanning, Guangxi, China; Cairo University Kasr Alainy Faculty of Medicine, EGYPT

## Abstract

**Background:**

Acute kidney injury (AKI) complicates the course of critical illness and carries high short- and long-term mortality rates; however, reliable early prognostic markers remain limited. The leuko-glycemic index (LGI), the product of white blood cell counts and blood glucose, has shown prognostic value in other acute conditions; however, its role in intensive care unit (ICU) patients with AKI remains unexplored.

**Methods:**

In this multicenter retrospective cohort study, we analyzed 112,235 adult ICU admissions from the MIMIC-IV (n = 54,840) and eICU-CRD (n = 57,395) databases. Patients with multiple ICU stays and those aged <18 years were excluded. The LGI was calculated within 24 h of admission and stratified into quartiles (Q1–Q4). The primary endpoints were 30- and 90-day in-hospital mortality, and the secondary endpoint was overall in-hospital mortality. Kaplan–Meier survival curves, multivariable Cox proportional hazards models, and restricted cubic splines (RCS) assess the association between LGI and outcomes, with subgroup and interaction analyses for key comorbidities.

**Results:**

Higher LGI quartiles were associated with progressively worse survival (p < 0.0001). In the fully adjusted models, each unit increase in LGI conferred a small but significant rise in 30-day (HR 1.01; 95% CI 1.01–1.01) and 90-day mortality (HR 1.01; 95% CI 1.01–1.01), while Q4 versus Q1 yielded HRs of 1.22 (95% CI 1.13–1.31) and 1.21 (95% CI 1.13–1.30), respectively. RCS demonstrated a nonlinear relationship, and the associations persisted across subgroups.

**Conclusion:**

LGI may be an independent biomarker associated with short-term mortality in ICU patients with AKI. Its potential clinical relevance warrants further investigation and validation in larger cohorts.

## 1. Introduction

Acute kidney injury (AKI) is clinically characterized by a sudden and significant decline in renal function, typically occurring over a short period of time, and represents one of the most prevalent and serious forms of organ dysfunction observed in critically ill patients [1,2]. AKI continues to pose a frequent and formidable challenge in medical practice, as it is closely associated with significantly higher mortality rates, extended durations of hospitalization, and an elevated risk of progression to chronic kidney disease over time [3,4]. Epidemiological data indicate that AKI affects approximately 10–15% of all patients admitted to hospitals for various reasons, with a prevalence exceeding 50% among patients requiring care in ICU, highlighting its substantial burden in acute care settings [1,5,6]. Despite ongoing advancements in supportive care and therapeutic interventions, the prognosis remains poor for individuals with severe AKI requiring kidney replacement therapy [7]. Therefore, it is necessary to explore indicators related to the prognosis of patients with AKI to improve their prognosis.

In 2010, Quiroga Castro et al. made a significant contribution to the field of cardiology by introducing the LGI, which has since been recognized as a valuable prognostic model for acute myocardial infarction [8]. In previous studies, LGI has been identified as an independent determinant of various diseases, including subarachnoid hemorrhage [9], coronary artery disease [10], and Acute Pulmonary Embolism [11].

This underscores the versatility and broad applicability of the LGI as a prognostic indicator across different disease spectra. However, despite these advances and the growing body of evidence supporting the utility of LGI in various clinical settings, a notable gap remains in our understanding of its prognostic significance in specific and critical patient populations. The prognostic significance of LGI in critically ill patients with AKI in the ICU remains uncertain, which has become a crucial area of interest and a priority for further investigations.

This uncertainty highlights the limitations of our current knowledge and emphasizes the need for more comprehensive and rigorous studies to establish its value as a prognostic biomarker. Given the high morbidity and mortality rates associated with AKI in ICU patients, identifying reliable and effective prognostic markers is of utmost importance for improving patient outcomes and guiding clinical decision-making. Against this backdrop, the present study has a clear and focused objective.

This study aimed to explore the complex and potentially significant association between the LGI and mortality in ICU patients with AKI. By employing robust research methodologies and analyzing relevant data from a diverse sample of patients, this study aimed to provide a more nuanced understanding of how LGI may serve as a prognostic biomarker in this specific patient group. Through careful examination and consideration of various factors that may influence this relationship, this study endeavors to offer valuable insights and evidence-based conclusions that could potentially enhance clinical practice and improve patient care in intensive care settings.

## 2. Materials

### 2.1. Data source

This comprehensive multicenter retrospective cohort study used extensive data derived from two highly reputable and extensively utilized medical databases in the field of critical care research, namely the Medical Information Mart for Intensive Care (MIMIC – IV) and eICU Collaborative Research Database (eICU - CRD) [12,13]. These databases are widely recognized for their robustness, comprehensiveness, and reliability in providing valuable information on intensive care patients, thereby offering a solid foundation for conducting large-scale and in-depth retrospective analyses.

This study utilized MIMIC-IV version 3.0, covering ICU admissions from 2008 to 2022, and the eICU-CRD (version 2.0), covering ICU admissions from 2014 to 2015.

The MIMIC-IV database was approved by the Institutional Review Boards of the Massachusetts Institute of Technology (Cambridge, MA, USA) and Beth Israel Deaconess Medical Center (Boston, MA, USA). The eICU-CRD database was approved by the Institutional Review Board of the Massachusetts Institute of Technology (Cambridge, MA, USA). Both databases contain de-identified patient data, and the requirement for individual patient consent was waived by the respective institutional review boards due to the retrospective nature of the study and the use of pre-existing, anonymized data. One of the authors of this study, Sheng Chen, successfully completed the Collaborative Institutional Training Initiative (CITI) program (record ID: 66963781) and obtained credentialed user status on PhysioNet, which is required for authorized access to these databases. Given that this was a retrospective study using publicly available, de-identified databases, informed consent was waived. We adhered to the Strengthening the Reporting of Observational Studies in Epidemiology (STROBE) guidelines

Given that this was a retrospective study and all patients were extracted from a public database, informed consent was waived. We adhered to the Strengthening the Reporting of Observational Studies in Epidemiology (STROBE) guidelines [14].

This study adhered to the ethical principles of the Declaration of Helsinki and followed the STROBE guidelines. Given the retrospective nature of the study and the use of de-identified data from publicly available databases, informed consent was waived. The MIMIC-IV database was approved by the Institutional Review Boards (IRB) of the Massachusetts Institute of Technology (IRB No. 0403000206) and Beth Israel Deaconess Medical Center (IRB No. 2001-P-001699/14).

For the eICU-CRD database, Institutional Review Board approval was not required due to its retrospective design and the absence of direct patient intervention. The database has undergone rigorous de-identification in accordance with the Safe Harbor provision of the US Health Insurance Portability and Accountability Act (HIPAA), removing all direct and indirect identifiers (including hospital and unit identifiers). Furthermore, the database architecture has been certified by privacy experts Priva Cert as compliant with Safe Harbor standards (HIPAA certification No. 1031219−2).

All patient data were de-identified, and patient privacy and confidentiality were maintained throughout the study in accordance with the respective Institutional Review Boards’ regulations.

### 2.2. Criteria for population selection

In this study, the research team focused on patients diagnosed with AKI and included them in the analysis. AKI is a common and serious condition in critically ill patients, and its occurrence can significantly affect patient outcomes, including increasing the risk of morbidity and mortality. Therefore, studying AKI is of great significance for improving the prognosis of critically ill patients. However, to ensure the robustness and specificity of the study population, several criteria were applied to exclude certain groups of patients. It is well known that pediatric populations often have distinct clinical characteristics and responses compared to adults. For example, their organ development, physiological functions, and responses to drugs and treatments may differ from those of adults. Therefore, patients aged < 18 years were excluded. Another factor that can introduce confounding effects is repeated hospitalization. When patients have multiple ICU admissions, the data from different admissions may be interrelated and influenced by previous hospitalization experiences, treatment, and disease progression. Thus, patients with multiple ICU admissions were excluded, and only data from the first admission were considered. By implementing these exclusion criteria, this study aimed to minimize potential biases and confounding factors, thereby enhancing the reliability and validity of the research findings.

### 2.3. Diagnostic criteria

AKI was the outcome, which was diagnosed based on Kidney Disease: Improving Global Outcomes Clinical Practice Guidelines [15]. People as an increase in SCr by ≥0.3 mg/dL (≥26.5 μmol/L) within 48 h, or an increase in SCr to ≥1.5 times the baseline, which is known or presumed to have occurred within the prior 7 days, or urine volume <0.5 mL/kg/h for 6 h.

### 2.4. Data collection

The baseline characteristics of patients within 24 h of ICU admission were meticulously extracted from the MIMIC-IV and eICU databases, which are renowned for their comprehensive and high-quality data in the field of critical care. These characteristics were categorized into five main domains to provide a holistic view of the patients’ conditions.

Demographic data, including age, sex, and other relevant personal information, provide essential background information for understanding patient profiles. Clinical severity scores, such as SOFA scores, were also included, as they serve as important indicators of the patients’ initial health status and the potential severity of their conditions upon ICU admission. Medication use was documented to track the types and dosages of drugs administered during the initial phase of the ICU stay, which can influence patient outcomes and interactions with other treatments.

Laboratory parameters, including vital signs and biochemical markers, provide insights into patients’ physiological states and organ functions. Comorbidities were recorded to account for any pre-existing conditions that might affect the patients’ responses to treatment and their overall prognosis. ICU treatment details were also extracted to understand the specific interventions and care protocols applied to patients during their critical care period.

Laboratory tests are crucial for assessing patients’ health status. Among these, white blood cell count (WBC, measured in ×10^9/L) and blood glucose level (measured in mmol/L) were specifically highlighted. WBC count is a key indicator of the body’s immune response and can reflect the presence of infection or inflammation. Blood glucose level is not only a marker of metabolic status but also has implications for various organ functions and the patients’ overall prognosis, especially in critically ill patients, where glucose metabolism can be significantly altered.

Drawing from the methodologies established in previous studies [9], the calculation of the LGI index was carefully conducted using the formula “WBC count (×10^9/L) multiplied by blood glucose levels (mmol/L)”. This formula combines two significant physiological parameters to create a composite index that may simultaneously reflect the inflammatory and metabolic status of patients.

To facilitate the analysis and interpretation of the data, participants were stratified into four quartiles (Q1, Q2, Q3, and Q4) based on their LGI values. This stratification approach allows for a more nuanced analysis of how varying levels of the LGI index may be associated with different outcomes. Q1, representing the lowest quartile of the LGI values, was designated as the reference group. This setup provides a benchmark against which the other quartiles can be compared, helping to identify potential trends or associations between the LGI values and the outcomes of interest in the study. By dividing the participants in this manner, this study can more effectively explore the potential prognostic value of the LGI index in the context of ICU patients with AKI.

### 2.5. Missing data

Multiple imputation was employed to address missing data for variables with less than 25% missing values [16].

### 2.6. Clinical outcomes

The primary endpoints were in-hospital 30- and 90-day mortality during the hospitalization period. The secondary endpoint was the all-cause mortality during hospitalization.

### 2.7. Statistical analysis

#### 2.7.1. Baseline comparisons and survival curves.

In this study, the statistical presentation of the data was carefully designed to accurately reflect the distribution and characteristics of the variables under investigation. Continuous variables, including laboratory parameters and demographic data, are presented as medians accompanied by interquartile ranges (IQR). This approach was chosen because it provides a robust measure of central tendency and dispersion, which is particularly suitable for data that may not follow a normal distribution, which is common in clinical datasets. Categorical variables, such as the presence or absence of specific comorbidities or demographic categories, are displayed as counts and percentages. This method allows for a clear and intuitive understanding of the proportions of different categories in the study population.

A combination of statistical tests was employed to compare the baseline characteristics across the different LGI quartile groups. Pearson’s chi-square tests or Fisher’s exact tests were utilized for categorical variables, depending on the sample size and the expected frequencies in the cells. These tests are standard methods for assessing associations between categorical variables and helped determine whether there were significant differences in the distribution of categorical variables, such as comorbidities, medication use, and demographic factors, across the LGI quartiles. The Kruskal-Wallis test was used for continuous variables. This non-parametric test does not assume a normal distribution of the data and is appropriate for comparing more than two independent groups, making it suitable for analyzing variables such as age, laboratory parameters, and clinical severity scores across the four LGI quartiles.

The Kaplan-Meier method, a well-established statistical approach in survival analysis, was employed to construct survival curves illustrating the probability of survival over time. These curves were generated for two critical time points: 30-day and 90-day mortality. The Kaplan-Meier estimator is particularly useful because it accounts for censored data, which in this context refers to patients who were lost to follow-up or discharged from the hospital before the specified time points. This method provides a visual and quantitative assessment of the survival experience of patients across different LGI quartiles, allowing preliminary exploration of the relationship between LGI and mortality outcomes.

#### 2.7.2. Multivariable Cox proportional hazards models.

To further investigate the association between LGI and all-cause in-hospital mortality in patients with AKI, multivariable Cox proportional hazards models were used. The Cox model is a powerful tool for survival analysis that allows for the simultaneous assessment of multiple variables and their potential confounding effects. It estimated the hazard ratio (HR) of mortality associated with LGI while adjusting for other relevant covariates. This approach helped isolate the independent effect of LGI on mortality, controlling for factors such as age, comorbidities, and treatment variables that might otherwise confound the relationship.

#### 2.7.3. Non-linear and subgroup analyses.

RCS were incorporated into the analysis to account for potential nonlinear relationships between the LGI and the primary outcomes of interest. Many biological relationships are not strictly linear, and the use of RCS allows for more flexible modeling of these associations. By fitting spline functions, the analysis can capture potential thresholds or inflection points in the relationship between LGI and mortality, providing a more accurate and nuanced understanding of how changes in the LGI might be associated with changes in risk.

Stratification and interaction analyses were conducted to evaluate how the association between LGI and mortality varied across different subgroups of the study population. Variables such as sex, age (categorized as ≤65 and >65 years), heart failure, respiratory failure, arterial fibrillation, stroke, and diabetes were considered in these analyses. Stratification involves dividing the dataset into subgroups based on these variables and examining the relationship between LGI and mortality within each subgroup. In contrast, interaction analyses statistically test whether the effect of LGI on mortality differs significantly between subgroups. Likelihood ratio tests were used to formally assess these interactions by comparing models with and without interaction terms to determine whether the inclusion of such terms significantly improved the model fit.

All statistical analyses were performed using R software, a widely recognized and validated platform for statistical computing and graphics, specifically version 4.4.0. A two-sided p-value threshold of less than 0.05 was adopted throughout the study to determine the statistical significance. This conventional threshold provides a standard benchmark for assessing the strength of evidence against the null hypothesis, helping ensure that the findings are not due to random chance. By adhering to these rigorous statistical methods and standards, this study aimed to provide reliable and valid insights into the role of LGI as a potential prognostic biomarker in ICU patients with AKI.

Each analytical step was pre-specified to address a distinct epidemiological question; no post-hoc statistical testing was performed.

## 3. Results

### 3.1. Demographics and Clinical Features

After meticulously applying the predefined exclusion criteria to ensure the homogeneity of the study population and the validity of the research, a substantial cohort of 112,235 patients was ultimately included in the comprehensive analysis. This extensive cohort was carefully assembled from two distinct sources, with 54,840 patients originating from the original cohort housed within the MIMIC-IV database, a well-established repository of critical care data. The remaining 57,395 patients were drawn from the validation cohort contained in the eICU-CRD, which serves as another vital source of information for intensivists and researchers worldwide.

**Fig 1:**
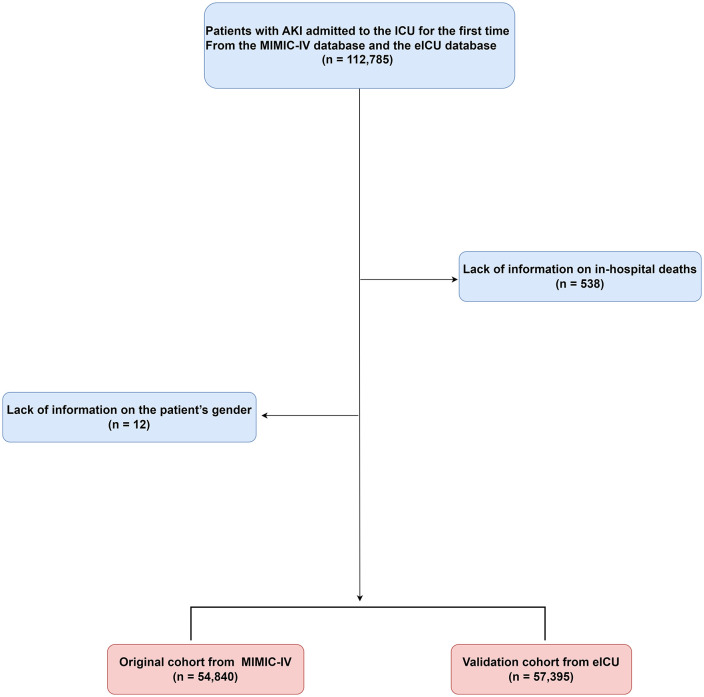
Flowchart of the study population.

For a clear and concise visualization of the patient selection process and the flow of participants through the study, refer to **Fig 1**. The figure illustrates the application of the inclusion and exclusion criteria, the sourcing of patients from the respective databases, and the final composition of the study cohort, providing transparency and facilitating the assessment of the study’s methodological rigor.

Furthermore, to offer an in-depth understanding of the study population’s characteristics and to establish a robust foundation for the subsequent analysis, the baseline characteristics of all included patients are meticulously detailed in **Table 1** and Supplementary **S1 Table**.

**Table 1:** Baseline characteristics of survivors and deceased patients in the original cohort.

Variable	Total (n = 54840)	Alive (n = 47545)	Death (n = 7295)	p.value
LGI	81.81(53.95,125.67)	79.32(53.03,119.94)	103.76(63.64,174.81)	<0.0001
Sex				<0.01
Female	23366(42.61)	20154(42.39)	3212(44.03)	
Male	31474(57.39)	27391(57.61)	4083(55.97)	
Age (years)	68.62(57.63,78.91)	68.05(57.00,78.26)	72.80(61.73,82.59)	<0.0001
AKI stage				<0.0001
1	16800(30.63)	15489(32.58)	1311(17.97)	
2	25361(46.25)	22958(48.29)	2403(32.94)	
3	12679(23.12)	9098(19.14)	3581(49.09)	
Weight (kg)	81.90(68.70,98.00)	82.60(69.40,98.80)	77.00(64.70,92.65)	<0.0001
Comorbidities				
Heart failure				<0.0001
No	37317(68.05)	32785(68.96)	4532(62.12)	
Yes	17523(31.95)	14760(31.04)	2763(37.88)	
Respiratory failure				<0.0001
No	37471(68.33)	34744(73.08)	2727(37.38)	
Yes	17369(31.67)	12801(26.92)	4568(62.62)	
Arterial fibrillation				<0.0001
No	36846(67.19)	32418(68.18)	4428(60.70)	
Yes	17994(32.81)	15127(31.82)	2867(39.30)	
Diabetes				0.04
No	35958(65.57)	31098(65.41)	4860(66.62)	
Yes	18882(34.43)	16447(34.59)	2435(33.38)	
Paraplegia				0.25
No	54521(99.42)	47261(99.40)	7260(99.52)	
Yes	319(0.58)	284(0.60)	35(0.48)	
Stroke				<0.0001
No	52087(94.98)	45282(95.24)	6805(93.28)	
Yes	2753(5.02)	2263(4.76)	490(6.72)	
Laboratory tests				
Hemoglobin, g/dL	10.54 ± 1.99	10.59 ± 1.97	10.23 ± 2.10	<0.0001
Platelet, K/uL	185.00(135.00,249.00)	187.00(138.00,249.00)	172.00(113.00,246.00)	<0.0001
RBC, m/uL	3.49(3.04,3.98)	3.50(3.07,3.99)	3.36(2.87,3.90)	<0.0001
WBC, K/uL	11.10(8.10,15.20)	10.90(8.00,14.80)	13.00(8.90,18.60)	<0.0001
SCr, mg/dL	1.10(0.80,1.70)	1.00(0.80,1.60)	1.60(0.90,2.70)	<0.0001
Glucose, mmol/L	7.17(5.94,9.11)	7.11(5.89,8.89)	7.94(6.28,10.67)	<0.0001
Sodium, mEq/L	138.80 ± 5.09	138.71 ± 4.86	139.34 ± 6.41	<0.0001
SOFA	4.00(2.00,7.00)	4.00(2.00,6.00)	8.00(5.00,11.00)	<0.0001
SAPSII	37.00(29.00,46.00)	35.00(28.00,43.00)	50.00(39.00,61.00)	<0.0001
OASIS	32.00(26.00,38.00)	31.00(26.00,37.00)	39.00(33.00,45.00)	<0.0001
CCI	5.00(3.00,7.00)	5.00(3.00,7.00)	7.00(5.00,9.00)	<0.0001
Drug use				
Epinephrine				<0.0001
No	52018(94.85)	45546(95.80)	6472(88.72)	
Yes	2822(5.15)	1999(4.20)	823(11.28)	
Dopamine				<0.0001
No	53194(97.00)	46498(97.80)	6696(91.79)	
Yes	1646(3.00)	1047(2.20)	599(8.21)	
Vasopressin				<0.0001
No	50483(92.06)	45307(95.29)	5176(70.95)	
Yes	4357(7.94)	2238(4.71)	2119(29.05)	

Data are presented Standard Deviation (SE) or frequencies (percentages).

Abbreviation: SOFA, sequential organ failure assessment; CCI, Charlson comorbidity index; SAPSII, simplified acute physiological score II; OASIS, oxford acute severity of illness score; WBC, white blood cell; RBC, red blood cell

In the original cohort, a notable observation was that a greater proportion of males were present in the death group than in the survival group. This sex disparity in mortality outcomes is an important demographic factor that may influence the overall prognosis and warrants further exploration in the context of AKI and other comorbid conditions. Furthermore, a clear association was observed between the severity of AKI and the risk of death. Specifically, the higher the AKI stage, the greater was the risk of death. This finding underscores the clinical significance of AKI staging in predicting adverse events.

When comparing various clinical and laboratory parameters between the death and survival groups, several key differences emerged. The death group exhibited significantly higher values for multiple indicators, including the LGI, WBC, serum creatinine (Scr), blood glucose, sodium, Sequential Organ Failure Assessment (SOFA) score, Simplified Acute Physiology Score II (SAPSII), Oxford Acute Severity of Illness Score (OASIS), and Charlson Comorbidity Index (CCI). These parameters are well-established markers of disease severity, organ dysfunction, and comorbidity burden. Their elevation in the death group suggests a more pronounced inflammatory response, metabolic derangement, and organ failure, which are critical determinants of mortality in critically ill patients with AKI.

Moreover, patients in the death group had a higher prevalence of several important comorbidities. Specifically, they were more likely to have heart failure, respiratory failure, atrial fibrillation, and stroke. These comorbidities are known to independently increase the risk of mortality and can interact with AKI to exacerbate the overall clinical condition.

Similar patterns and associations were consistently observed in the validation cohort, which strengthened the validity and generalizability of these findings. The replication of results across two distinct cohorts suggests that these factors are robust predictors of mortality in patients with AKI and are not merely artifacts of a specific dataset or patient population. This consistency is crucial for establishing the clinical relevance and applicability of the study’s conclusions in diverse critical care.

### 3.2. Associations between LGI and patient outcomes

#### 3.2.1. Kaplan–Meier survival analysis curves for mortality.

**Fig 2:**
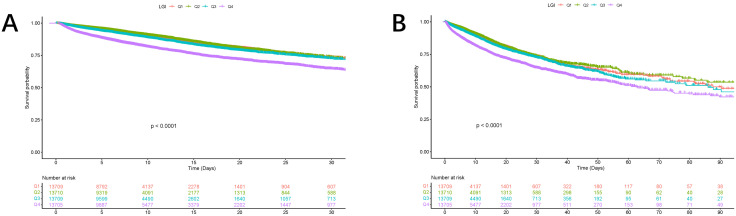
Kaplan–Meier curves showing the cumulative probability of mortality. (A) Death within 30 days in the original cohort, (B) within 90 days in the original cohort, (C) within 30 days in the validation cohort, and (D) within 90 days in the validation cohort.

The Kaplan – Meier survival analysis, a widely recognized statistical method for estimating survival probabilities over time, is comprehensively presented in **Fig 2**. This figure serves as a dynamic visual tool that illustrates survival outcomes based on stratification by the LGI quartile, offering an intuitive understanding of how the LGI correlates with patient prognosis in the context of AKI.

In the original cohort, the analysis revealed significant differences in in-hospital mortality at two critical time points: 30 days (**Fig 2A**) and 90 days (**Fig 2B**). These time points were chosen because they represent important milestones in the short-term recovery and management of critically ill patients with AKI. Among the four patient groups categorized according to their LGI quartiles, the survival curves clearly diverged, indicating varying risks of mortality across the groups. The statistical significance of these differences was rigorously assessed and confirmed with a p-value of less than 0.0001, providing robust evidence of the association between LGI values and mortality risk.

The analysis clearly demonstrated that the group with the highest LGI exhibited a substantially higher risk of mortality than that of the group with the lowest LGI. This finding is particularly striking as it suggests a dose-response relationship, where increasing LGI values are linked to progressively poorer patient outcomes. This pattern not only highlights the potential of the LGI as a strong predictor of short-term mortality but also implies that it could serve as a valuable tool for risk stratification and prognosis assessment in the clinical management of patients with AKI.

Similar trends were observed in the validation cohort (**Fig 2C**, **2D**), where the survival curves again showed significant differences in mortality across the LGI quartiles. The consistency of these findings across two distinct cohorts strengthens the validity and generalizability of the results, suggesting that the LGI index holds promise as a reliable prognostic biomarker in diverse populations of ICU patients with AKI. This is further supported by the statistically significant associations observed in both cohorts, reinforcing the notion that LGI could be integrated into clinical practice to aid decision-making and improve patient care.

#### 3.2.3. Cox Proportional Hazard Ratios for All-Cause Mortality.

Cox proportional hazards analysis, a powerful statistical tool widely used in survival analysis to assess the association between one or more predictor variables and time-to-event outcomes, was meticulously conducted to examine the relationship between LGI and in-hospital mortality, with the results presented in **Table 2**.

**Table 2:** Cox regression analysis of LGI and mortality in patients with AKI.

Categories	crude model		Model 1		Model 2		Model 3	
95%CI	P	95%CI	P	95%CI	P	95%CI	P
30-day in-hospital mortality								
Continuous variable per unit	1.01(1.01,1.01)	<0.0001	1.01(1.01,1.01)	<0.0001	1.01(1.01,1.01)	<0.0001	1.01(1.01,1.01)	<0.0001
Quartile								
Q1	ref		ref		ref		ref	
Q2	0.95(0.88,1.03)	0.22	0.96(0.89,1.04)	0.30	0.99(0.92,1.07)	0.80	1(0.92,1.08)	0.99
Q3	1.16(1.08,1.25)	<0.0001	1.19(1.11,1.28)	<0.0001	1.08(1.00,1.16)	0.05	1.09(1.01,1.17)	0.03
Q4	1.86(1.74,1.99)	<0.0001	1.94(1.81,2.07)	<0.0001	1.24(1.16,1.32)	<0.0001	1.22(1.13,1.31)	<0.0001
p for trend		<0.0001		<0.0001		<0.0001		<0.0001
90-day in-hospital mortality								
Continuous variable per unit	1.01(1.01,1.01)	<0.0001	1.01(1.01,1.01)	<0.0001	1.01(1.01,1.01)	<0.0001	1.01(1.01,1.01)	<0.0001
Quartile								
Q1	ref		ref		ref		ref	
Q2	0.94(0.87,1.02)	0.12	0.95(0.88,1.02)	0.18	0.98(0.91,1.06)	0.65	1(0.92,1.07)	0.91
Q3	1.16(1.08,1.24)	<0.0001	1.18(1.10,1.27)	<0.0001	1.08(1.00,1.16)	0.05	1.09(1.01,1.17)	0.03
Q4	1.8(1.69,1.92)	<0.0001	1.88(1.76,2.00)	<0.0001	1.22(1.14,1.31)	<0.0001	1.21(1.13,1.30)	<0.0001
p for trend		<0.0001		<0.0001		<0.0001		<0.0001

Crude model: unadjusted

Model 1: adjusted for sex, age, weight

Model 2: adjusted for sex, age, weight, CCI, OASIS, SAPS II, SOFA

Model 3: adjusted for sex, age, weight, CCI, OASIS, SAPS II, SOFA, Sodium, Serum creatinine, RBC, Platelet, Hemoglobin, Stroke, Paraplegia, Arterial fibrillation, Respiratory failure, Heart failure, diabetes, Epinephrine, Dopamine, Vasopressin

Abbreviation: SOFA, sequential organ failure assessment; CCI, Charlson comorbidity index; SAPSII, simplified acute physiological score II; OASIS, oxford acute severity of illness score; WBC, white blood cell; RBC, red blood cell

In the original cohort, when focusing on 30 – day mortality, the analysis revealed compelling evidence of LGI’s prognostic value of LGI. When treating LGI as a continuous variable, which allows for the assessment of the effect of a one-unit increase in LGI on mortality risk, multiple models consistently identified it as a significant risk factor for mortality. This indicates that even a small increase in LGI may be associated with a higher risk of death within 30 days. In the fully adjusted model, which meticulously controlled for a comprehensive set of potential confounders, such as age, comorbidities, and other relevant clinical variables, the HR was determined to be 1.01. The 95% confidence interval (CI) was narrow, ranging from 1.01 to 1.01, and the result was highly statistically significant (p < 0.0001). This suggests a stable and precise estimate of effect size.

When evaluating LGI as a categorical variable, which divides the cohort into groups based on LGI tertiles and compares the highest tertile to the lowest tertile, the analysis again demonstrated a significant association. Patients in the highest tertile of LGI faced a substantially higher risk of 30 – day mortality compared to those in the lowest tertile. In the fully adjusted model, the HR was 1.22, indicating a 22% increase in the risk of mortality for those in the highest tertile. The 95% CI of 1.13–1.31 further reinforces the consistency and reliability of this finding, with the p-value remaining highly significant at less than 0.0001.

Similar analyses were performed for 90 – day mortality, extending the time frame to capture long-term outcomes. When LGI was treated as a continuous variable, the results paralleled those of the 30 – day analysis. Multiple models have identified LGI as a significant risk factor for mortality. In the fully adjusted model, the HR was 1.01, with a 95% CI of 1.01–1.01 and a p-value < 0.0001, indicating that the association between LGI and mortality risk persisted beyond the initial 30 – day period.

For the categorical analysis of 90 – day mortality, the highest tertile of LGI continued to show a significantly higher risk of mortality than the lowest tertile. In the fully adjusted model, the HR was 1.21, reflecting a 21% increase in mortality risk. The 95% CI of 1.13–1.30 and p-value < 0.0001 provided further confirmation of the robustness of this association over the 90 – day period.

The validation cohort yielded analogous findings (**S2 Table**), reinforcing the consistency and generalizability of the results across datasets. This strengthens the argument for LGI’s potential of LGI as a reliable prognostic biomarker for in-hospital mortality in patients with AKI. The concordance of results across the original and validation cohorts suggests that the observed associations are not dataset-specific and are likely to be clinically relevant in diverse populations of ICU patients with AKI.

#### 3.2.4. RCS for all-cause mortality.

**Fig 3:**
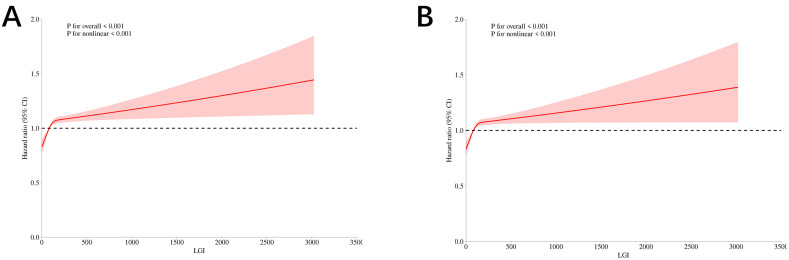
RCS for all-cause mortality. (A) 30-day mortality in the original cohort. (B) 90-day mortality in the original cohort. (C) 30-day mortality in the validation cohort. (D) 90-day mortality in the validation cohort.

In the original cohort (**Fig 3A**, **3B**), the LGI demonstrated a nonlinear association with both 30-day in-hospital mortality in patients with AKI (p < 0.001) and 90-day in-hospital mortality (p < 0.001).

In the validation cohort (**Fig 3C**, **3D**), the LGI exhibited a nonlinear relationship with 30-day (P for nonlinear = 0.026) and 90-day in-hospital mortality (P for nonlinear = 0.030).

### 3.3. Subgroup analysis

**Table 3** presents a stratified analysis of the relationship between LGI and mortality in the original cohort. This analysis delved deeper into the data by examining how the LGI index performed across different subgroups of patients, thereby offering a more nuanced understanding of its predictive capabilities. The results consistently highlight the significant predictive value of LGI for in-hospital mortality across various patient groups, reinforcing its potential as a versatile biomarker in the complex landscape of critical care medicine.

**Table 3:** HRs for all-cause mortality in the different subgroups of the original cohort.

Character	HR (95% CI)	p	p for interaction
Age			0.124
>65	1.002(1.002,1.003)	<0.0001	
≤65	1.003(1.002,1.003)	<0.0001	
Sex			0.937
Female	1.003(1.002,1.003)	<0.0001	
Male	1.003(1.002,1.003)	<0.0001	
Heart failure			0.359
Yes	1.003(1.002,1.003)	<0.0001	
No	1.002(1.002,1.003)	<0.0001	
Respiratory failure			< 0.0001
Yes	1.001(1.001,1.002)	<0.0001	
No	1.003(1.003,1.003)	<0.0001	
Arterial fibrillation			0.315
Yes	1.002(1.002,1.003)	<0.0001	
No	1.003(1.002,1.003)	<0.0001	
Stroke			0.109
Yes	1.003(1.002,1.004)	<0.0001	
No	1.003(1.002,1.003)	<0.0001	
Diabetes			< 0.0001
Yes	1.002(1.002,1.002)	<0.0001	
No	1.003(1.003,1.004)	<0.0001	

A higher LGI was associated with an increased risk of death in patients with AKI, indicating that this composite index of WBC count and blood glucose levels captures critical aspects of the patients’ physiological stress and inflammatory response. This association suggests that the LGI can serve as a valuable tool for identifying patients at a higher risk of adverse outcomes, which is crucial for prioritizing interventions and allocating healthcare resources effectively.

The results showed a similar trend in the validation cohort (**S3 Table**). The consistency of the findings across both the original and validation cohorts strengthens the argument for the robustness and generalizability of the LGI as a prognostic marker. This cross-cohort validation is essential for building confidence in the clinical utility of the LGI and paves the way for its potential integration into routine clinical practice for risk assessment in patients with AKI.

## 4. Discussion

In this retrospective, multicenter cohort study, we comprehensively evaluated the potential relationship between the LGI and mortality outcomes among patients diagnosed with AKI who were admitted to the ICU. By examining this association, we aimed to determine whether the LGI could serve as a reliable and clinically meaningful indicator of prognosis in this critically ill patient group. Our findings clearly demonstrate that the LGI, which is calculated based on the interaction between peripheral WBC count and blood glucose concentration, acts as an independent prognostic factor for predicting mortality in ICU patients with AKI. This suggests that LGI may reflect underlying systemic inflammation and metabolic stress, both of which are crucial contributors to adverse outcomes in critical care. This study highlights the potential clinical utility of LGI as a simple, cost-effective, and readily available biomarker for stratifying the risk of both short-term and long-term mortality in this high-risk patient population.

A major strength of this investigation lies in its robust multicenter design, which incorporates data from two large-scale, well-validated, and widely used critical care databases: the MIMIC-IV and eICU-CRD. These datasets encompass a broad spectrum of critically ill patients across multiple hospital settings, enhancing the external validity and applicability of our findings. The inclusion of both a development and an independent validation cohort significantly increased the methodological rigor and credibility of the study, ensuring that the observed associations were not merely dataset-specific artifacts. Notably, the consistent association between LGI and mortality observed in both the original and validation cohorts provides strong evidence to support the robustness and reproducibility of our results. This cross-cohort consistency further underscores the reliability of LGI as a prognostic indicator of mortality among ICU patients with AKI and highlights its potential as a valuable tool for clinical decision-making and risk assessment in this vulnerable population.

Our analysis, conducted using the Kaplan-Meier survival curve methodology, revealed distinct and statistically significant differences in in-hospital mortality rates when patients were stratified according to the quartiles of the LGI. Specifically, patients in higher LGI quartiles exhibited a markedly increased risk of death during hospitalization compared with those in lower quartiles, highlighting a clear dose-response relationship between elevated LGI and adverse outcomes. This association was consistently observed across both 30-day and 90-day mortality endpoints, reinforcing the prognostic significance of LGI and suggesting its potential role as an early and reliable indicator of short-and intermediate-term mortality risk. These findings underscore the clinical relevance of LGI in the early identification of critically ill patients at a heightened risk of poor outcomes.

Importantly, our study results align with and support a growing body of literature in which elevated LGI values have been associated with unfavorable prognoses in various acute medical conditions, including acute myocardial infarction and subarachnoid hemorrhage [8,9]. These studies provide a contextual foundation that lends credibility to our observations and suggest that LGI may reflect a shared pathophysiological mechanism involving inflammation and metabolic dysregulation across different disease states. Furthermore, our research contributes novel and clinically meaningful insights by being one of the first studies to explore and establish the prognostic value of LGI in the specific setting of AKI among critically ill patients. This adds an important dimension to the understanding of LGI, emphasizing its applicability beyond traditional cardiovascular and neurological contexts and extending its potential utility to nephrology and intensive care medicine.

The LGI is a composite parameter that combines the inflammatory indicator leukocyte count with the metabolic marker of glycemic control derived from blood glucose levels to provide a single index reflective of both systemic inflammation and metabolic dysregulation [17]. The rationale behind the LGI lies in the recognition that hyperglycemia and leukocytosis are closely interrelated physiological responses that can exacerbate tissue injury, drive endothelial dysfunction, and promote prothrombotic states in various acute and chronic conditions [17].

A growing number of clinical studies have rigorously assessed the clinical significance of LGI in the context of acute myocardial infarction and various forms of acute coronary syndromes. In particular, among patients who present with ST‐elevation myocardial infarction, elevated LGI levels at the time of admission have been consistently linked to a range of adverse clinical outcomes, including higher rates of in‐hospital mortality, an increased incidence of heart failure, and a greater likelihood of experiencing major adverse cardiovascular events during hospitalization [18]. These findings suggest that LGI may serve as a valuable marker of heightened inflammatory and metabolic stress in ST‐elevation myocardial infarction, with important implications for early risk stratification and management strategies.

In addition to its role in acute coronary syndromes, LGI has also been explored in patients with coronary chronic total occlusion, a condition characterized by complete blockage of a coronary artery for an extended period of time. In these patients, particularly among elderly individuals, higher LGI values have been shown to independently predict the presence of multivessel coronary artery disease, indicating a greater overall burden of atherosclerosis [19,20]. Furthermore, an elevated LGI in this population has been associated with worse long-term clinical outcomes, including increased rates of recurrent ischemic events and all-cause mortality. These observations highlight the prognostic relevance of LGI not only in acute settings but also in chronic ischemic cardiovascular disease, reinforcing its potential utility as a broadly applicable biomarker for identifying patients at increased cardiovascular risk across a spectrum of clinical scenarios.

The kidneys play a significant role in maintaining glucose homeostasis through various mechanisms, including gluconeogenesis, glucose uptake, and reabsorption [21]. The underlying basis of renal injury is thought to be impaired energetics in highly metabolically active nephron segments, such as the proximal tubules and the thick ascending limb in the renal outer medulla [22]. This can trigger a conversion from transient hypoxia to intrinsic renal failure. Kidney cell injury can be lethal or sublethal, with the latter influencing GFR and renal blood flow [22].

The role of hyperglycemia in kidney injury remains debatable. Some studies have suggested that hyperglycemia may directly contribute to kidney injury. In diabetic mouse models, renal ischemia-reperfusion induced more severe AKI and higher mortality, and the severity correlated with blood glucose levels [23]. In vitro, high glucose-conditioned renal proximal tubular cells show higher apoptosis and caspase activation following injury [23].

The relationship between WBC count and the incidence of AKI has been investigated in several studies. A U-shaped relationship was found between WBC count and AKI risk in critically ill patients [24]. In a study of 2,079 ICU patients, both leukopenia and leukocytosis were associated with an increased risk of AKI and mortality [24]. The 1st and 5th quintiles of WBC counts had greater odds ratios for AKI (1.42 and 2.05, respectively) and mortality (1.40 and 1.36, respectively) than the 3rd quintile [24].

Our multivariable Cox proportional hazards regression models provided robust confirmation of the strong and statistically significant association between the LGI and all-cause mortality in patients with AKI. Specifically, the analysis showed that individuals with elevated LGI values exhibited a substantially higher risk of mortality, and this relationship remained consistent for both the 30-day and 90-day survival periods. Importantly, this association persisted even after adjusting for a comprehensive set of potential confounding variables, including demographic factors, comorbidities, and laboratory parameters, underscoring the independent prognostic value of LGI in critically ill patients with AKI. These findings suggest that LGI has the potential to serve as a clinically useful and independent biomarker for mortality risk assessment, offering prognostic information beyond that provided by traditional indicators.

The nonlinear association between LGI and mortality, as illustrated by the application of RCS in our statistical modeling, further highlights the complexity of the relationship between this biomarker and patient outcomes. The RCS analysis revealed a nonlinear risk curve, indicating that the prognostic implications of LGI vary at different levels and that its predictive utility may be particularly pronounced in patients with extremely elevated LGI values. This nonlinear pattern suggests that LGI could be especially helpful in identifying individuals at the highest risk of death and may inform more personalized clinical decision-making strategies in the ICU setting.

Our subgroup analysis further emphasized the robustness and consistency of the LGI as a mortality predictor across various clinically relevant patient subgroups. Notably, the LGI maintained its prognostic significance among patients with different comorbid conditions, including heart failure, respiratory failure, and diabetes, suggesting its broad applicability in heterogeneous clinical populations. This subgroup consistency reinforces the potential of the LGI as a versatile risk stratification tool that remains reliable even when patient characteristics differ significantly. The presence of such diverse patient profiles in both the original and validation cohorts enhances the generalizability of our findings and reflects the real-world heterogeneity typically encountered in ICU populations.

Despite the strengths and clinical implications of our study, it is important to acknowledge several limitations that should be considered when interpreting our results. First, as a retrospective observational study, our analysis was based on data collected during routine clinical care rather than through a controlled experimental protocol, which introduces the possibility of bias and unmeasured confounding. Second, although the MIMIC-IV and eICU-CRD databases are among the most comprehensive critical care datasets available and include detailed clinical and laboratory data, they may still lack certain relevant variables that could influence patient prognosis and might have enhanced the model performance if included. Finally, although we employed multiple imputation techniques to address the issue of missing data—assuming that data were missing at random—there remains a risk of residual confounding due to unmeasured or systematically missing variables, which could affect the validity of our conclusions.

The original and validation cohorts span different and partially non-overlapping time periods. However, the LGI is derived from standardized laboratory measurements (WBC and glucose) that reflect objective pathophysiological states rather than practice-dependent variables. The consistency of the LGI–mortality association across both databases supports the robustness of our findings despite temporal differences. As a retrospective study using publicly available, de-identified databases, patient and public involvement was not possible. Future prospective studies are needed to validate the clinical utility of LGI-guided risk stratification at the bedside.

## 5. Conclusion

This multicenter retrospective cohort study, involving large and diverse patient populations from multiple hospitals and regions, showed a significant correlation between the LGI and both short- and long-term mortality in ICU patients with AKI. Higher LGI values were significantly associated with increased 30-day and 90-day mortality in Kaplan-Meier survival analysis and Cox proportional hazards models. The consistency of these findings across varied clinical settings and patient subgroups highlights the robustness, generalizability, and clinical relevance of LGI as a simple biomarker for mortality risk in critically ill patients with AKI. Further prospective studies are required to confirm its clinical utility in routine practice.

## Supporting information

S1 Table Baseline characteristics of survivors and deceased patients in the Validation cohort. (DOCX)

S2 Table Cox regression analysis of LGI and mortality in patients with AKI in Validation cohort. (DOCX)

S3 Table HRs for all-cause mortality in different subgroups in the Validation cohort. (DOCX)

## Abbreviations

AKI: Acute kidney injury
ICU: intensive care unit
LGI: leuko-glycemic index
ICU: intensive care unit
RCS: restricted cubic splines
MIMIC – IV: Medical Information Mart for Intensive Care
eICU – CRD: eICU Collaborative Research Database
WBC: white blood cell count
Scr: serum creatinine
SOFA: Sequential Organ Failure Assessment
SAPSII: Simplified Acute Physiology Score II
OASIS: Oxford Acute Severity of Illness Score
CCI: Charlson Comorbidity Index
HR: hazard ratio
