# Supplementary material for: Association of leuko-glycemic index with mortality in ICU patients with Acute kidney injury: A retrospective multicenter cohort study
Source: PLoS One. 2026 Jun 4;21(6):e0350811. doi: 10.1371/journal.pone.0350811 (PMC13235893; doi:10.1371/journal.pone.0350811)
Supplement: S1 Table — (DOCX) [file pone.0350811.s001.docx]

**S1 Table.** Baseline characteristics of survivors and deceased patients in the Validation cohort.

| Variable | Total (n=57395) | Alive (n=8774) | Death (n=48621) | p.value |
| --- | --- | --- | --- | --- |
| LGI | 107.90(66.58,178.64) | 136.40(78.40,241.73) | 104.17(65.09,168.89) | <0.0001 |
| Sex |  |  |  | 0.38 |
| Female | 25931(45.18) | 4002(45.61) | 21929(45.10) |  |
| Male | 31464(54.82) | 4772(54.39) | 26692(54.90) |  |
| Age (years) | 65.30 ± 15.91 | 69.09 ± 14.78 | 64.61 ± 16.01 | <0.0001 |
| Weight(kg) | 86.74 ± 28.27 | 83.30 ± 28.63 | 87.36 ± 28.16 | <0.0001 |
| AKI score |  |  |  | <0.0001 |
| 1 | 23734(41.35) | 3074(35.04) | 20660(42.49) |  |
| 2 | 17637(30.73) | 2265(25.81) | 15372(31.62) |  |
| 3 | 16024(27.92) | 3435(39.15) | 12589(25.89) |  |
| Comorbidities |  |  |  |  |
| Respiratory failure |  |  |  | <0.0001 |
| No | 41495(72.30) | 4781(54.49) | 36714(75.51) |  |
| Yes | 15900(27.70) | 3993(45.51) | 11907(24.49) |  |
| Heart failure |  |  |  | <0.001 |
| No | 50459(87.92) | 7607(86.70) | 42852(88.13) |  |
| Yes | 6936(12.08) | 1167(13.30) | 5769(11.87) |  |
| Arterial fibrillation |  |  |  | <0.0001 |
| No | 51041(88.93) | 7491(85.38) | 43550(89.57) |  |
| Yes | 6354(11.07) | 1283(14.62) | 5071(10.43) |  |
| Diabetes |  |  |  | <0.0001 |
| No | 49342(85.97) | 7678(87.51) | 41664(85.69) |  |
| Yes | 8053(14.03) | 1096(12.49) | 6957(14.31) |  |
| Paraplegia |  |  |  | 0.72 |
| No | 57319(99.87) | 8764(99.89) | 48555(99.86) |  |
| Yes | 76(0.13) | 10(0.11) | 66(0.14) |  |
| Stroke |  |  |  | <0.0001 |
| No | 54001(94.09) | 8047(91.71) | 45954(94.51) |  |
| Yes | 3394(5.91) | 727(8.29) | 2667(5.49) |  |
| Laboratory tests |  |  |  |  |
| SAPSII | 35.00(26.00,46.00) | 50.00(38.00,64.00) | 33.00(25.00,43.00) | <0.0001 |
| SOFA | 7.00(5.00,9.00) | 10.00(7.00,13.00) | 6.00(5.00,9.00) | <0.0001 |
| CCI | 4.00(2.00,6.00) | 5.00(3.00,7.00) | 4.00(2.00,6.00) | <0.0001 |
| OASIS | 28.00(22.00,36.00) | 37.00(29.00,45.00) | 27.00(21.00,34.00) | <0.0001 |
| WBC, K/uL | 12.30(8.79,17.20) | 12.00(8.60,16.60) | 14.50(9.75,20.90) | <0.0001 |
| RBC, m/uL | 3.88 ± 0.80 | 3.80 ± 0.83 | 3.89 ± 0.80 | <0.0001 |
| Glucose, mmol/L | 8.44(6.67,11.39) | 8.33(6.61,11.11) | 9.33(7.00,13.17) | <0.0001 |
| Hemoglobin, g/dL | 11.63 ± 2.36 | 11.43 ± 2.45 | 11.66 ± 2.34 | <0.0001 |
| Platelet, K/uL | 204.00(151.00,269.00) | 197.00(133.00,268.00) | 205.00(153.00,269.00) | <0.0001 |
| Sodium, mEq/L | 139.47 ± 5.52 | 140.23 ± 6.76 | 139.33 ± 5.25 | <0.0001 |
| SCr, mg/dL | 1.38(0.91,2.45) | 1.82(1.17,2.90) | 1.30(0.90,2.30) | <0.0001 |
| Drug use |  |  |  |  |
| Vasopressin |  |  |  | <0.0001 |
| No | 53356(92.96) | 6808(77.59) | 46548(95.74) |  |
| Yes | 4039(7.04) | 1966(22.41) | 2073(4.26) |  |
| Dopamine |  |  |  | <0.0001 |
| No | 53977(94.04) | 7804(88.94) | 46173(94.97) |  |
| Yes | 3418(5.96) | 970(11.06) | 2448(5.03) |  |
| Epinephrine |  |  |  | <0.0001 |
| No | 53600(93.39) | 7605(86.68) | 45995(94.60) |  |
| Yes | 3795(6.61) | 1169(13.32) | 2626(5.40) |  |

Data are presented Standard Deviation (SE) or frequencies (percentages).

Abbreviation: SOFA, sequential organ failure assessment; CCI, Charlson comorbidity index; SAPSII, simplified acute physiological score II; OASIS, oxford acute severity of illness score; WBC, white blood cell; RBC, red blood cell
