# Supplementary material for: Association of leuko-glycemic index with mortality in ICU patients with Acute kidney injury: A retrospective multicenter cohort study
Source: PLoS One. 2026 Jun 4;21(6):e0350811. doi: 10.1371/journal.pone.0350811 (PMC13235893; doi:10.1371/journal.pone.0350811)
Supplement: S2 Table — (DOCX) [file pone.0350811.s002.docx]

**S2 Table.** Cox regression analysis of LGI and mortality in patients with AKI in Validation cohort.

| Categories | crude model | | Model 1 | | Model 2 | | Model 3 | |
| --- | --- | --- | --- | --- | --- | --- | --- | --- |
|  | 95%CI | P | 95%CI | P | 95%CI | P | 95%CI | P |
| 30-day in-hospital mortality |  |  |  |  |  |  |  |  |
| Continuous variable per unit | 1.01(1.01,1.01) | <0.0001 | 1.01(1.01,1.01) | <0.0001 | 1.01(1.01,1.01) | <0.0001 | 1.01(1.01,1.01) | <0.0001 |
| Quartile |  |  |  |  |  |  |  |  |
| Q1 | ref |  | ref |  | ref |  | ref |  |
| Q2 | 1.02(0.96,1.10) | 0.51 | 1.01(0.95,1.09) | 0.72 | 1.03(0.96,1.10) | 0.47 | 1.03(0.96,1.11) | 0.38 |
| Q3 | 1.17(1.09,1.25) | <0.0001 | 1.17(1.09,1.25) | <0.0001 | 1.02(0.96,1.09) | 0.50 | 1.04(0.97,1.11) | 0.31 |
| Q4 | 1.87(1.76,1.99) | <0.0001 | 1.92(1.81,2.04) | <0.0001 | 1.28(1.20,1.36) | <0.0001 | 1.28(1.20,1.37) | <0.0001 |
| p for trend |  | <0.0001 |  | <0.0001 |  | 0.01 |  | 0.002 |
| 90-day in-hospital mortality |  |  |  |  |  |  |  |  |
| Continuous variable per unit | 1.01(1.01,1.01) | <0.0001 | 1.01(1.01,1.01) | <0.0001 | 1.01(1.01,1.01) | <0.0001 | 1.01(1.01,1.01) | <0.0001 |
| Quartile |  |  |  |  |  |  |  |  |
| Q1 | ref |  | ref |  | ref |  | ref |  |
| Q2 | 1.01(0.95,1.09) | 0.67 | 1(0.94,1.07) | 0.91 | 1.02(0.96,1.09) | 0.53 | 1.03(0.96,1.10) | 0.42 |
| Q3 | 1.16(1.08,1.23) | <0.0001 | 1.16(1.08,1.23) | <0.0001 | 1.02(0.96,1.09) | 0.57 | 1.03(0.97,1.10) | 0.33 |
| Q4 | 1.82(1.72,1.93) | <0.0001 | 1.87(1.76,1.98) | <0.0001 | 1.27(1.19,1.35) | <0.0001 | 1.27(1.20,1.36) | <0.0001 |
| p for trend |  | <0.0001 |  | <0.0001 |  | 0.02 |  | 0.002 |

Crude model: unadjusted

Model 1: adjusted for sex, age, weight

Model 2: adjusted for sex, age, weight, CCI, OASIS, SAPS II, SOFA

Model 3: adjusted for sex, age, weight, CCI, OASIS, SAPS II, SOFA, Sodium, Serum creatinine, RBC, Platelet, Hemoglobin, Stroke, Paraplegia, Arterial fibrillation, Respiratory failure, Heart failure, diabetes, Epinephrine, Dopamine, Vasopressin

Abbreviation: SOFA, sequential organ failure assessment; CCI, Charlson comorbidity index; SAPSII, simplified acute physiological score II; OASIS, oxford acute severity of illness score; RBC, red blood cell
