# Supplementary material for: Association of leuko-glycemic index with mortality in ICU patients with Acute kidney injury: A retrospective multicenter cohort study
Source: PLoS One. 2026 Jun 4;21(6):e0350811. doi: 10.1371/journal.pone.0350811 (PMC13235893; doi:10.1371/journal.pone.0350811)
Supplement: S3 Table — (DOCX) [file pone.0350811.s003.docx]

**S3 Table.** HRs for all-cause mortality in different subgroups in the Validation cohort.

| Character | HR (95% CI) | p | p for interaction |
| --- | --- | --- | --- |
| Age |  |  | < 0.0001 |
| >65 | 1.002(1.002,1.002) | <0.0001 |  |
| ≤65 | 1.001(1.001,1.001) | <0.0001 |  |
| Sex |  |  | 0.123 |
| Female | 1.002(1.001,1.002) | <0.0001 |  |
| Male | 1.001(1.001,1.001) | <0.0001 |  |
| Heart failure |  |  | 0.578 |
| No | 1.001(1.001,1.002) | <0.0001 |  |
| Yes | 1.002(1.001,1.002) | <0.0001 |  |
| Arterial fibrillation |  |  | 0.805 |
| No | 1.001(1.001,1.002) | <0.0001 |  |
| Yes | 1.001(1.001,1.002) | <0.0001 |  |
| Respiratory failure |  |  | 0.003 |
| No | 1.001(1.001,1.002) | <0.0001 |  |
| Yes | 1.001(1.001,1.001) | <0.0001 |  |
| Stroke |  |  | < 0.0001 |
| No | 1.001(1.001,1.002) | <0.0001 |  |
| Yes | 1.003(1.003,1.004) | <0.0001 |  |
| Diabetes |  |  | < 0.0001 |
| No | 1.002(1.002,1.002) | <0.0001 |  |
| Yes | 1.001(1.000,1.001) | <0.0001 |  |
